# Feed intake and dietary composition of iron (Fe), copper (Cu), vitamin E, and tannic acid of five captive black rhinoceros (*Diceros bicornis*) in a UK collection

**DOI:** 10.1002/zoo.21580

**Published:** 2020-11-02

**Authors:** Victoria Ricketts, Ellen S. Dierenfeld, Cathrine Sauer, Katherine Whitehouse‐Tedd

**Affiliations:** ^1^ School of Animal, Rural and Environmental Sciences Nottingham Trent University Southwell Nottinghamshire UK; ^2^ Ellen S. Dierenfeld, LLC St. Louis Missouri USA; ^3^ Department of Science Chester Zoo Chester UK; ^4^ Copenhagen Zoo Frederiksberg Denmark

**Keywords:** browser, captive feeding, herbivore, iron overload, nutrition

## Abstract

The black rhinoceros (*Diceros bicornis*) is a critically endangered species facing multiple anthropogenic pressures in its natural home range across Africa. Black rhinoceros are difficult to maintain ex situ and subject to diseases that are linked with captive dietary factors. Hemochromatosis is of particular concern, as it is a common finding at necropsy of captive adults, and has been linked to excessive dietary iron intake. This intake study investigates the select nutrient composition of the diets offered to and consumed by five captive black rhinoceros in a UK zoo to evaluate, ensure adequacy, and/or make adjustments if necessary. Alfalfa hay, pellets and six browse species offered were analyzed for iron (Fe), copper (Cu), vitamin E, and tannic acid content. Intakes were quantified and evaluated against levels found in wild diets and the currently available feeding guidelines for black rhinoceros. Diets eaten by five individual rhinoceros (1.4%–2.3% of bodyweight dry matter [DM] intake), comprising 68%–82% hay, 6%–13% pellets, and 13%–27% browse, contained 76–98 mg/kg Fe (on a DM basis), fell within the ranges of plants eaten by free‐ranging rhinoceros (45–140 mg/kg DM), as well as values recommended for captive‐fed browsing rhinoceros (50–100 mg/kg DM). Consumed diets were found to be marginal to adequate in Cu (9–11 mg/kg DM) compared with the recommended 10 mg/kg DM; dietary vitamin E ranged from 54 to 79 IU/kg DM, and tannic acid measured 13–14 g/kg DM. Commercial pellets were the primary contributor of dietary Fe, Cu, and vitamin E, containing up to 10 times more of each of those nutrients than the forages. Native browses were important sources of lower Fe ingredients, as well as appropriate levels of dietary Cu and vitamin E (dependent on species). Interestingly, pellets (23 g/kg) and alfalfa hay (14 g/kg) contained higher concentrations of tannic acid compared with any of the browses fed (4–13 g/kg). All nutritional parameters evaluated were close to recommended dietary levels, diets resembled values consumed in the wild, and the animals remained clinically healthy throughout the study. Overall, diets were considered nutritionally adequate for captive feeding of black rhinoceros; evaluating the nutrient composition of all ingredients, including browse plants in diets, provides important information for achieving optimal nutrient balance.

## Research Highlights

Local browse plants contained higher Cu and vitamin E levels, but lower Fe and tannic acid compared with alfalfa fed to black rhinoceros and were considered important forages for meeting dietary nutrients required by this endangered browser.

## Graphical abstract

Dietary iron (Fe) and copper (Cu) intakes in black rhinoceros in a UK zoo (black bars) compared with free‐ranging (white bars) and feeding recommendations (gray bars).

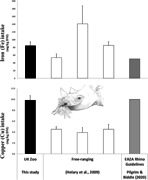

## 1 STATEMENT OF PROBLEM

As a browsing species, the black rhinoceros (*Diceros bicornis*) is considered difficult to maintain ex situ due to difficulties in meeting their nutritional needs (Clauss & Dierenfeld, 2008). This species is prone to diseases that can be linked with dietary factors in captivity (Clauss & Dierenfeld, 2008; Clauss & Hatt, 2006; Sullivan & Valdes, 2019), many of which are not present in wild counterparts (Clauss et al., 2012; Helary et al., 2009). Deposition of iron (Fe) in multiple organs of the animal is of particular concern, as it has been a common finding at necropsy of adults in captivity (Molenaar et al., 2008; Paglia & Tsu, 2012). Previous studies of nutrient intake of captive browsing rhinoceros reported findings of high dietary Fe in conjunction with copper (Cu) deficiencies (Dierenfeld et al., 2000, 2006). Low vitamin E concentrations have also been associated with captive diseases in this species; most notably, low vitamin E status has been suspected as a factor underlying the occurrence of hemolytic anemia (Dierenfeld et al., 1988; Sullivan et al., 2015). It has also been hypothesized that diets fed to captive black rhinoceros lack naturally occurring iron‐binding chelators, such as tannins, compared with wild diets (Clauss, 2003; Helary et al., 2009). The objectives of this investigation were: (1) to perform an intake study to determine consumed levels of selected dietary chemical constituents (Fe, Cu, vitamin E, and tannic acid) by captive black rhinoceros in a UK zoological collection and (2) to compare values with the European Association of Zoos and Aquariums (EAZA) husbandry guidelines for this species and with previously published studies on free‐ranging browsing rhinoceros.

## 2 DESCRIPTION OF PROCESS

An intake study was conducted with five adult black rhinoceros (2.3) at Chester Zoo in accordance with the British and Irish Association of Zoos and Aquariums (BIAZA) guidelines (Bishop et al., 2013) and ethical approval was granted by the School of Animal, Rural and Environmental Sciences ethics review group (ARE580), Nottingham Trent University. Over a single 5‐ or 4‐day period, individual rhinos were housed and fed in separate pens and paddocks as per their standard husbandry routines, which consisted of one daily pelleted feed on the hardstand near the house and of two‐three feeds of alfalfa hay and browse spread out on the paddock. Alfalfa and browse were always offered in different locations to avoid mixing of leftovers; pellets were always completely consumed. Diets offered to the rhinoceros consisted of 20–25 kg (one to one and a half bale) of alfalfa (*Medicago sativa*) hay, 750–3000 g of a commercial herbivore pellet (Zebra Cube; Dodson and Horrell), and as much browse as possible. For logistical reasons, the study had to be carried out over two consecutive weeks in August 2017 (2.1 individuals in the first week, 0.2 in the next). The females studied in the latter week were both lactating and housed with their suckling‐only calves. Animals remained clinically healthy and exhibited normal behaviors throughout the investigation. Each individual feed item in the diet was weighed daily to the nearest 0.1 kg before being offered to each rhinoceros. The following morning, the remaining leftovers of each feed item were collected by hand, visually sorted, and weighed back. Browses offered were locally sourced. Quantities and species of browse offered (alder, common [*Alnus glutinosa*]; ash, common [*Fraxinus excelsior*]; birch, silver [*Betula pendula*]; oak, common [*Quercus robur*]; willow, goat [*Salix caprea*]; willow, white [*S. alba*]) varied according to daily availability. On days where multiple species of browse were offered, the different types were separated for weighing; both branches and leaves were offered but parts were not weighed separately.

Composite samples of each diet item were collected as follows: alfalfa hay (~1 kg) was sampled by collecting four representative grab samples from three different bales stored in three separate locations at the zoo housing black rhinoceros. Similarly, a sample of the pellets (~100 g) was taken from three different bags at the same feed storage locations. Browse samples (~500 g) were collected from the daily deliveries and included leaves, branches of varying diameter, and any other organic matter, for example, acorns, as rhinos consumed all parts of the browses offered. All samples were stored in labeled vacuum‐sealed plastic bags and kept refrigerated (5–10°C) for 4–6 weeks as vitamin E degradation in fresh plant materials is reduced under refrigeration compared with freezing (Dierenfeld, unpublished data). At the end of the study period, daily samples of each species of browse were pooled for analysis. All feed samples were shipped by overnight courier on ice to Sciantec Analytical (a division of Cawood Scientific Ltd.). In the laboratory, the samples were dried in a drying oven at 60°C and ground to pass through a 1 mm screen. The ground feed samples were then analyzed for Fe and Cu using ICP‐OES (S1015 a UKAS accredited procedure). Vitamin E was determined by high‐performance liquid chromatography with UV/Vis and fluorescence detection (S1181). Tannic acid concentrations were determined using colorimetry (S1166). Results of laboratory analysis are found in Table 1.

**Table 1:** Chemical composition of feed items offered to five black rhinoceros (*Diceros bicornis*) in a UK institution

Feed item	Dry matter (%)	Iron (Fe) (mg/kg)^a^	Copper (Cu) (mg/kg)^a^	Vitamin E (α‐tocopherol) (IU/kg)^b^	Tannic acid (g/kg)
Alfalfa hay	89	60	7.0	19.3	13.5
*Medicago sativa*		(120)	(6.0)		
Pellets	92	378 (341)	36 (40)	372.5	23.1
Alder, common *Alnus glutinosa*	47	47.0	4.0	120.1	4.3
Ash, common *Fraxinus excelsior*	54	72.0	21.0	16.7	8.4
Birch, silver *Betula pendula*	47	44.0	8.0	77.8	3.8
Oak, common *Quercus robur*	51	57.0	8.0	152.0	13.0
Willow, goat *Salix caprea*	54	47.0	11.0	163.1	10.4
Willow, white *Salix alba*	49	43.0	12.0	124.2	7.3

*Note*: Values (except dry matter) are on a dry matter basis.

^a^ Values presented in parentheses are from feed analysis carried out by the institution the year before during the same season, using the same analytical methods by the same laboratory, Sciantec Analytical, Selby, UK.

^b^ Vitamin E value is based on leaves and twigs analyzed together as presented/consumed by the rhinoceros.

For the determination of dry matter (DM) intake, duplicate samples of each feed item were taken both before being offered and from leftovers for all individual rhinoceros every day of the trials. These samples were also kept refrigerated in vacuum‐sealed plastic bags for 2–3 weeks before being dried to constant weight at 100°C. Average DM percentages for each feed item offered and leftover were used to convert fresh weights to offered and leftover DM to determine daily DM intake (DMI).

## 3 DEMONSTRATION OF EFFICACY

DM intake for the five rhinoceros in this study ranged from 1.4% to 2.3% of bodyweight, as seen in Table 2. Hay contributed the most to the overall diet with 67.6%–82.0% of DMI, then browse (12.5%–26.6% DMI), followed by pellets contributing the least (5.5%–12.7% DMI). The five rhinoceros consumed between 74% and 92% (DM) of hay offered, 39%–58% (DM) of the browse offered, and pellets were consumed in their entirety every day. On average, 42% (range: 38%–54%) of the nutrients investigated in this study were contributed from hay, 37% (range: 29%–50%) from pellets and 21% (range: 14%–35%) from browse.

**Table 2:** Bodyweights and average daily diet consumption (kg), presented on both a dry matter (DM) and as fed (AF) basis, and % of bodyweight for five black rhinoceros *(Diceros bicornis)* in a UK institution

Rhino ID	Male 1^a^	Male 2	Female 1^a^	Female 2	Female 3
Body mass, kg	1004	1073	1245	1068	1121
Daily DMI, % body mass	2.0	2.1	1.4	2.1	2.3
Alfalfa hay eaten, kg DM	15.2	15.5	11.7	16.2	21.0
Pellets eaten, kg DM	1.9	1.9	1.0	2.8	1.4
Browse eaten, kg DM	3.2	5.2	4.6	3.0	3.2
Alfalfa hay eaten, kg AF	16.3	14.9	12.4	–b	22.4
Pellets eaten, kg AF	2.0	2.0	1.0	3.0	1.5
Browse eaten, kg AF	6.2	7.3	9.6	7.5	7.3

Abbreviation: DMI, dry matter intake.

^a^ Values based on four 24‐h periods of intake data.

^b^ Due to soaking, following a weighing of offered alfalfa hay, AF consumption could not be determined from fresh weights.

Based on the analysis and amounts of foods eaten (Tables 1 and 2, respectively), calculated consumed dietary concentrations of Fe, Cu, vitamin E, and tannic acid are presented in Table 3.

**Table 3:** Average select nutrient concentrations in total diets consumed by five black rhinoceros (*Diceros bicornis*) in a UK institution

Dietary nutrient concentration	Male 1^a^	Male 2	Female 1^a^	Female 2	Female 3	Rhino dietary values^b^ (min)
Fe (mg/kg)^c^	87.9 (129.0)	83.0 (120.0)	75.6 (113.3)	98.3 (138.0)	76.4 (123.5)	50
Cu (mg/kg)^c^	10.0 (9.6)	10.1 (9.7)	9.4 (9.1)	11.0 (10.6)	9.0 (8.4)	10
Vitamin E^d^ (IU/kg)	72.1	79.0	72.9	78.3	53.7	150–200^e^
Tannic acid (g/kg)	13.9	13.5	13.3	14.1	13.7	n/a

*Note*: All nutrients on a dry matter basis.

^a^ Values are based on four 24‐h periods of intake data.

^b^ Pilgrim and Biddle (2020).

^c^ Values in parentheses were calculated using analytical data from previous samples of alfalfa hay and pelleted feed used at this institution, as presented in Table 1.

^d^ Measured as α‐tocopherol.

^e^ Suggested dietary vitamin E concentration for lactating females.

**Figure 1:**
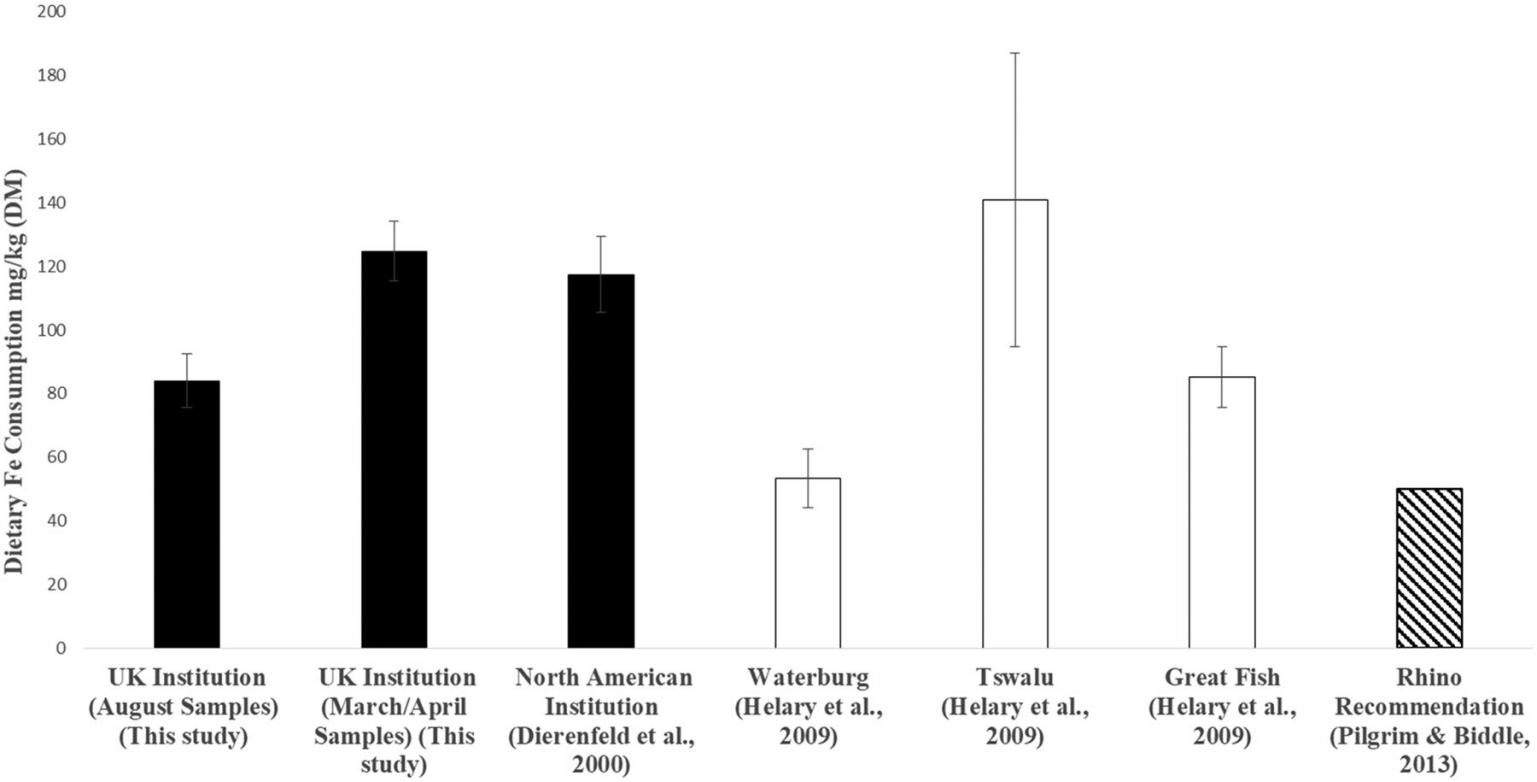
Comparison of mean dietary Fe concentrations (mg/kg dry matter) for five black rhinoceros (*Diceros bicornis*) in a UK institution (August and March/April samples) (this study), three Sumatran rhinoceros (*Dicerorhinus sumatrensis*) in a North American institution, and three free‐ranging populations of black rhinoceros (South Africa) versus black rhinoceros diet recommendation

In this collection, the mean quantified dietary Fe concentration of 84.2 ± 8.4 (range 76–98, Table 3) was very close to the high end of the recommended range (50–100 mg/kg) dietary Fe targets (all DM basis) determined for this species (minimum: Pilgrim & Biddle, 2020; maximum 300: Clauss et al., 2012). Additionally, our data compares favorably with dietary Fe concentrations consumed by free‐ranging black rhinoceros sampled from three African locations (45–140 mg/kg DM, Helary et al., 2009; see Figure 1). Given the high Fe content analyzed in the pellets (and higher values measured in alfalfa sampled from this institution the previous year [120 mg/kg DM; Table 1]), a dietary minimum of 50 mg/kg DM Fe may be difficult to achieve, and a target value of ~100 mg/kg DM dietary Fe may be a more realistic recommendation for browsers in captivity being offered a practical diet of hays and pelleted feeds (Clauss et al., 2012; Mylniczenko et al., 2012). Pelleted feeds often contain high Fe content, likely due to a combination of high background Fe in raw ingredients, as well as (often) addition of supplemental Fe in trace mineral premixes. Iron can also be contributed directly through milling equipment; noniron‐based equipment can reduce the iron content of final products. A recommendation that manufactured products fed to browsing rhinoceros in captivity contain ≤300 mg/kg DM Fe (Clauss et al., 2012) may only be achieved through the use of lower Fe ingredients—such as locally available browse plants—and/or in combination with the elimination of supplemental Fe added to mineral premixes, or the use of noniron‐containing manufacturing equipment. Nonetheless, these actions should be considered important for the long‐term health of captive browsing rhinoceros. Based on the data from this study, another option might be to offer forages only (hays, browses) as sole feedstuffs for these species to meet the dietary goal of Fe concentrations <100 mg/kg DM, if adequate supplies are available and diets can be nutritionally balanced.

**Figure 2:**
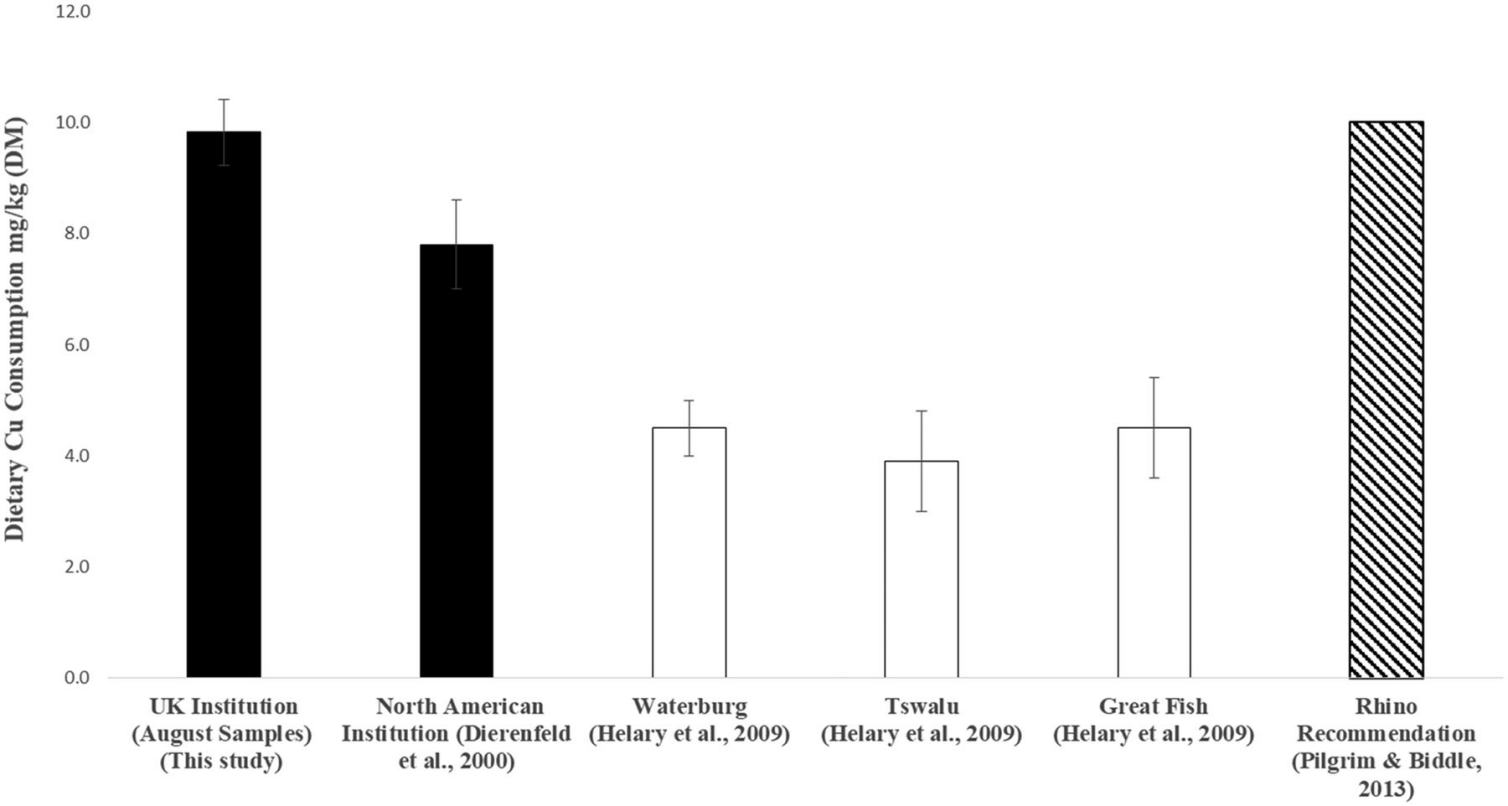
Comparison of mean dietary Cu concentrations (mg/kg dry matter) for five black rhinoceros (*Diceros bicornis*) in a UK institution (August samples; this study), three Sumatran rhinoceros (*Dicerorhinus sumatrensis*) in a North American institution, and three free‐ranging populations of black rhinoceros (South Africa) versus black rhinoceros diet recommendation

Cu intake was, on average, marginally adequate in this collection (9.8 ± 0.6 mg/kg DM [range: 9–11]) when compared with the rhinoceros dietary minimum recommendations of 10 mg/kg DM (Table 3). However, the current diet is closely aligned to values reported from three free‐ranging populations of black rhinoceros in Africa (3.5–4.5 ± 0.9 mg/kg DM, Helary et al., 2009; Figure 2), along with data measured in other captive browser rhinoceros in North America (range: 6.9–8.2 ± 0.8; Dierenfeld et al., 2000). Helary et al. (2009) suggest that as their three free‐ranging populations showed no reported signs of Cu deficiency, low Cu intake does not appear to be a limiting factor for health and/or reproduction in the groups they studied. Thus, dietary Cu concentrations <10 mg/kg DM for captive animals may not be absolutely deficient (Wichert et al., 2002). Similarly, Dierenfeld et al. (1995) measured Cu concentrations between 3 and 12 mg/kg DM in 26 browse plants consumed by free‐ranging black rhinoceros in Zimbabwe. Nonetheless, Pagan (1998) suggests that, in particular, reproducing equids require higher dietary copper—up to 30 mg/kg DM—to maintain optimal status, especially if consuming diets high in protein and/or calcium, as may be found with alfalfa as the primary forage. This may be of relevance to feeding captive browser rhinoceros, and warrants further investigation, as marginal copper status in captive browser rhinoceros was previously suggested from tissue mineral analyses in a larger, multispecies study (Dierenfeld et al., 2005). Finally, potential Cu/Fe interactions have not been fully explored in browsing rhinoceros and may add further understanding to trace mineral nutrition.

Diets fed to the five rhinoceros in this study contained vitamin E levels averaging 71.2 ± 9.2 IU/kg DM (range 54–79 IU/kg; Table 3) and were generally higher than EAZA rhinoceros minimum recommendations of 50 IU/kg DM (Pilgrim & Biddle, 2020, based on established equine dietary requirements for maintenance (National Research Council, 2007). However, they represent low values compared with recently revised dietary recommendations (Pilgrim & Biddle, 2020; Sullivan & Valdes, 2019) of 150–200 IU vitamin E per kg DM, originally proposed by Dierenfeld (1999). Female 3, who had a suckling calf, consumed a diet containing the lowest vitamin E concentration (Table 3). It is also worth noting that Female 3 had the lowest intake of Cu within this collection, possibly due to her consuming the greatest proportional intake of hay, which is low in both Cu and vitamin E. Only two of six browse species offered contained quantified concentrations of vitamin E between 150 and 200 IU/kg DM; nonetheless, overall browse ranges for this nutrient compare favorably to 26 freshly sampled browses eaten by rhinoceros in Zimbabwe (~10–286 IU/kg DM; Dierenfeld et al., 1995). The browse species containing the highest vitamin E content (goat willow; 163 IU/kg DM) was also the most frequently offered/consumed species during the study period. Considering that all five rhinoceros diets in this study and only 33% (2/6) of offered species of browse plants met the current guideline values established for black rhinoceros (150–200 IU/kg DM; Pilgrim & Biddle, 2020), vitamin E supplementation may be required to adequately meet nutritional needs in this collection. Other measures of vitamin E status, such as tissue concentrations, would be required to confirm health status; further, it is possible that the stable vitamin E added in the manufactured pellets may be an important contributing dietary source. However, browse vitamin E concentrations measured in this study must be considered minimal estimates of vitamin E activity. Storage (4–6 weeks) and drying before analysis likely oxidized vitamin E such that the actual amounts consumed by the rhinoceros fed browse on a daily basis would be expected to be higher. Nonetheless, as has been reported in the literature (Record et al., 1989; Wilson, 1987), excess Fe stores can place an increased demand on vitamin E reservoirs for its antioxidant capacity. Considering the unknown Fe and total iron‐binding capacity status of this UK collection, further investigation (plasma sampling) is warranted to determine vitamin E status for this group (Dierenfeld et al., 1988). Also, an iron panel, including transferrin saturation, ferretin, and serum iron could better elucidate nutrient interactions (Sullivan & Valdes, 2019).

Tannic acid concentrations in consumed diets averaged 13.7 ± 0.3 g/kg (range 13–14 g/kg DM) during this study, or about 1% of DM (Table 3), which is substantially lower than the 5% tannic acid or quebracho tannins fed to rhinos in another study (i.e., Clauss et al., 2005) that demonstrated induction of salivary‐binding proteins. Dietary tannic acid levels between ~5% and 20% of DM have been shown to significantly reduce Fe absorption in model species (rats, Afsana et al., 2004; pigs, Lee et al., 2010; fruit bats, Lavin et al., 2010). Additionally, tannic acid concentrations reported here were lower than condensed tannin ranges reported in native browses consumed by free‐ranging black rhinoceros (3.0 ± 1.0% of DM; Helary et al., 2012). Hence, not only should dietary concentrations, but also tannin types, be considered in evaluating dietary effects on iron bioavailability. An iron‐binding tannin analysis, instead of simply tannin content, may provide a more relevant measure for diet assessment in rhinoceroses and other species prone to iron overload syndromes (see Lavin et al., 2015; Lavin, 2012). Hay contributed the most total tannic acid in diets during this study, but surprisingly the pellets contained the highest measured concentration; typically, hay and pellets are not considered good sources of dietary tannins (Clauss et al., 2005, 2006). The potential source of tannic acid in the pellets is unknown. Tannic acid content of the browses fed during this study (3.8%–13.0% of DM) were higher than soluble tannin concentrations in 17 browses found in the natural diet of black rhinoceros (0.2%–4.1% of DM; Loutit, 1987). However, previous studies have also found that tannic acid content can differ widely from species to species and even between plant parts, as well as be influenced by environmental and growing conditions (Elgailani & Ishak, 2014). Regardless, these tannic acid data represent some of the first data of their kind reported for diets of captive rhinoceros fed practical (rather than experimental) diets.

It may be necessary to identify more detailed guidelines, including minimum and maximum ranges, particularly for Fe and Cu concentrations, in conjunction with an evaluation of Fe serum levels to properly assess captive nutrient status in black rhinoceros. Continued investigations of vitamin E concentrations and tannins in browses (both amounts and types) and other feed items being offered in captivity should also be considered to better understand nutrient interactions and effects on animal health, with standardized methodologies recommended for comparisons among facilities, as well as in situ environments.

## Data Availability

Raw data are available by contacting the first author.
